# Alcohol Dysregulates Corticotropin-Releasing-Hormone (CRH) Promoter Activity by Interfering with the Negative Glucocorticoid Response Element (nGRE)

**DOI:** 10.1371/journal.pone.0026647

**Published:** 2011-10-24

**Authors:** Magdalena M. Przybycien-Szymanska, Natasha N. Mott, Toni R. Pak

**Affiliations:** Department of Cell and Molecular Physiology, Stritch School of Medicine, Loyola University Chicago, Maywood, Illinois, United States of America; INSERM, UMR-S747, France

## Abstract

EtOH exposure in male rats increases corticotropin-releasing hormone (CRH) mRNA in the paraventricular nucleus of the hypothalamus (PVN), a brain region responsible for coordinating stress and anxiety responses. In this study we identified the molecular mechanisms involved in mediating these effects by examining the direct effects of EtOH on CRH promoter activity in a neuronal cell line derived from the PVN (IVB). In addition, we investigated the potential interactions of EtOH and glucocorticoids on the CRH promoter by concomitantly treating cells with EtOH and the glucocorticoid receptor (GR) antagonist RU486, and by sequentially deleting GR binding sites within glucocorticoid response element (GRE) on the CRH promoter. Cells were transiently transfected with a firefly luciferase reporter construct containing 2.5 kb of the rat wild type (WT) or mutated CRH promoter. Our results showed that EtOH treatment induced a biphasic response in CRH promoter activity. EtOH exposure for 0.5 h significantly decreased promoter activity compared to vehicle treated controls, whereas promoter activity was significantly increased after 2.0 h of EtOH exposure. Treatment with RU486, or deletion of the GR binding sites 1 and 2 within the GRE, abolished the EtOH-induced increase in the promoter activity, however did not affect EtOH-induced decrease in CRH promoter activity at an earlier time point. Overall, our data suggest that alcohol exposure directly regulates CRH promoter activity by interfering with the normal feedback mechanisms of glucocorticoids mediated by GR signaling at the GRE site of the CRH promoter.

## Introduction

Alcohol is a potent activator of the hypothalamo-pituitary adrenal (HPA) axis, as manifested by immediate increases in circulating glucocorticoids following exposure [1], [2], [3], [4], [5]. Although the effects of alcohol on HPA function have been well described, our understanding of the molecular mechanisms regulating alcohol effects on the HPA axis remain poorly defined. Corticotrophin-releasing hormone (CRH)-expressing neurons located in the paraventricular nucleus of the hypothalamus (PVN) play a pivotal role in orchestrating the central stress response and proper functioning of these neurons is critical for maintaining a homeostatic state following a stressful event. The HPA axis is a three-tiered biological system that begins at the highest level with CRH release from the PVN potentiating the release of adrenocorticotrophin hormone (ACTH) from the anterior pituitary gland. ACTH acts, in turn, on the adrenal glands to increase the production and release of glucocorticoid hormones [6]. Glucocorticoids (CORT) can then exert negative feedback on both the hypothalamus and pituitary gland to decrease CRH and ACTH release [6], [7], [8].

We previously demonstrated that binge-pattern alcohol exposure during pubertal development increased both circulating plasma CORT levels and CRH mRNA expression in the PVN [4], suggesting that alcohol exposure disrupted normal glucocorticoid negative feedback pathways. Glucocorticoid negative feedback is mediated, in part, by the activation of glucocorticoid receptors (GR), which belong to the superfamily of nuclear steroid receptors. Upon activation by glucocorticoids, GRs undergo dimerization, translocate to the nucleus, and modulate gene transcription [9], [10], [11], [12]. In the PVN, GRs are known to decrease CRH gene transcription through signaling at the negative glucocorticoid response element (nGRE), located between −249 and −278 nucleotides upstream from the transcription start site of the CRH promoter. Overall, glucocorticoids acting through GRs decrease CRH promoter activity thereby, decreasing transcriptional activity of the promoter and decreasing CRH gene expression.

Based on our previous observations that binge-pattern EtOH exposure in pubertal rats increased CRH gene expression in the PVN [4], we tested the hypothesis that ethanol (EtOH) increases CRH gene expression by directly interfering with glucocorticoid negative feedback at the level of the CRH promoter. The overall goals of this study were to determine if 1) EtOH directly modulates CRH promoter activity and 2), to identify a putative site of action for EtOH on the CRH promoter. Overall, our results showed that EtOH differentially modulated CRH promoter activity in a time-dependent manner. Further, these effects were mediated, in part, through the nGRE site on the CRH promoter. Taken together, our data provide strong evidence that EtOH exposure directly disrupts GR:CRH signaling which, if occurs during adolescence, may be detrimental for proper maturation of the HPA axis.

## Materials and Methods

### Cell Culture

The IVB cell line, derived from the rat hypothalamic PVN, was used for all transient transfections (generously provided by Dr. John Kaskow, University of Cincinnati) and was verified to be free of mycoplasma contamination (data not shown, MycoSensor QPCR, Stratagene/Agilent Technologies). Cells were maintained in DMEM containing 4.5% glucose and L-glutamine (HyClone Laboratories, Logan, UT) supplemented with 10% fetal bovine serum. Cells were grown to 90% confluence and all transient transfections were performed within 10 passages.

### Reporter gene constructs and expression vectors

The full-length rat CRH promoter was generously provided and validated by Dr. Audrey F. Seasholtz (University of Michigan, Ann Arbor, MI) and then modified as follows. The full- length promoter fragment (−2125/+94) was excised from the pUC18 vector by restriction enzyme digestion for EcoR1 (5′) and HINDIII (3′) and subsequently subcloned into the promoterless luciferase vector (pGL3 basic, Promega Corp., Madison, WI). The pRL-tk-luciferase reporter vector (Promega Corp., Madison, WI) was used as an internal control for calculating plasmid transfection efficiency.

### Transient Transfections and Dual Luciferase Assay

Cells were plated at the density of 20, 000 cells/well in opaque 96-well plate for 24 hours prior to transfection to achieve a final confluency of 70–90%. Transient transfections were performed in replicates of 6 wells/plate for each construct/treatment and each assay was repeated minimum of 6 times (N = 6). Transfections were achieved using a lipid-mediated transfection reagent, Fugene6 (Roche Molecular Biomedical, Indianapolis, IN) according to manufacturer's instructions. Twenty-four hours following transfection, cells were treated (see below) and then processed for luciferase assays (Dual Luciferase Reporter (DLR) kit (Promega Inc., Madison, WI). Briefly, cells were lysed in 20 µl of lysis buffer, incubated on a shaker for 20 min at room temperature, and then loaded into a multiple well plate reader (Synergy HT, Biotech). The plate reader is equipped with dual injectors and automatically dispenses 100 µl firefly luciferase substrate (LARII) followed by “stop-and glo” substrate for renilla luciferase. Results were analyzed using Gen5 software (Biotech Inc., Winooski, VT).

### EtOH and Forskolin Treatments

Twenty-four hours after transfection with the CRH promoter, cells were incubated with varying concentrations of EtOH (12.5, 25.0, 50.0 or 100 mM) diluted in 10% FBS media (vehicle) for 2.0 h (dose response experiments), or they were treated with 12.5 mM EtOH for either 0.5, 1.0, 2.0, 4.0 h (time course studies). Cells transfected with the mutated CRH promoter that lacked specific GR regulatory regions were treated with 12.5 mM EtOH or vehicle for 0.5, 1.0, 2.0 or 4.0, or 8 h or, in control experiments, they were treated with 25 µM Forskolin for 6.0 h.

### Kinase Inhibitors

The following kinase inhibitors were purchased from Sigma Aldrich (St. Louis, MO) and used at a concentration of 10 µM each: LY 294,002 (PI3K inhibitor), SB 202190 (p38 MAPK inhibitor), and H89 (PKA inhibitor). Twenty-four hours after transfection (as described above), cells were exposed to concomitant treatment with kinase inhibitors and 12.5 mM EtOH, or kinase inhibitor alone, for 0.5, 1.0, or 2.0 h.

### Site-Directed Mutagenesis

**Figure 1:**
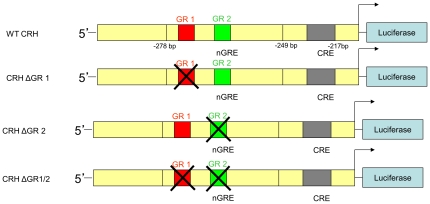
Schematic representation of mutant CRH promoter constructs. Diagrams depicting specific nucleotide sequences deleted within the nGRE site of the CRH promoter located between −249 and −278 bp upstream from the transcription initiation site (arrow).

Scanning mutagenesis deletions were performed on the CRH promoter using the QuickChange II XL kit according to manufacturer's instructions (Stratagene, LaJolla, CA). Briefly, forward and reverse primer sequences were designed targeting the appropriate identified regions of the promoters (GR binding site 1, (5′-CTTGGATAATCTCATTCAAGAACAATGGACAAGTCATAAGAGC-3′; 5′-GAACCTATTAGAGTAAGTTCTTGTTACCTGTTCAGTATTCTTCG-3′), GR binding site 2 (5′-CTCATTCAAGAATTTTTGTCAACAAGTCATAAGAAGCCCTTCCA-3′; 5′-GAGTAAGTTCTTAAAAACAGTTGTTCAGTATTCTTCGGGAAGGR-3′), and GR binding site 1 and 2 double deletion (5′-GGATAATCTCATTCAAGAACAAAGTCATAAGAAGCCCTTCCA-3′; 5′TGGAAGGGCTTCTTATGACTTTGTTCTTGAATGAGATTATCC-3′. Sequences contained deletions of 7, 4, or 6 bp, respectively, sequentially from 5′ to 3′ in these regions (Fig. 1). A standard PCR reaction was performed on a thermal cycler using mutated CRH-luciferase construct as a template. Following the reaction, the parent plasmid was digested using the DpnI restriction enzyme and the daughter plasmid, containing the desired mutation was transformed into XL-10 Ultragold competent cells and amplified. The mutation was confirmed by DNA sequencing using the in-house core sequencing facility (Loyola University Chicago, Stritch School of Medicine).

### qRT-PCR

GR gene expression levels were measured in IVB cells after EtOH treatment. Cells were plated at the density of 200,000 cells/well in clear 6-well plate until they achieved final confluence of 90%. At that time (approximately 72.0 h later) cells were treated with 100 mM EtOH or vehicle for 0.5 or 2.0 h. After appropriate treatment times, cells were washed 2 times with cold PBS and 1 ml of Trizol was added to each well. Total RNA isolation was performed on sonicated samples using Trizol reagent (Invitrogen Inc., Carlsbad, CA) according to the manufacturer's directions. Following RNA isolation, 0.5 µg total RNA was reverse transcribed using the First Strand Synthesis SuperMix for qRT-PCR (Invitrogen Inc., Carlsbad, CA). Roche FastStart SYBR Green Master Mix was added to GR specific upper and lower primer (0.25 µM final concentration; 5-AAACCTCAATAGGTCGACCAGCGT; 5-AGGTGCTTTGGTCTGTGGGATACA). Then, 2 µL cDNA templates were added to duplicate reactions performed in 96 well plates. Quantification of the target gene expression was achieved by extrapolating from standard curve of known concentrations of the hypoxanthine guanine phosphoribosyl transferase 1 (HPRT) housekeeping gene ran simultaneously in the same plate. All samples were normalized to the HPRT housekeeping gene, as it is not altered by EtOH treatment [4].

### Western Blot Analysis

Cells at a density of 10^5^ cells/dish were treated with vehicle (10% FBS media) or 100 mM EtOH for 0.5 or 2.0 h. Total protein was isolated using Tissue Protein Extraction Reagent (Thermo Scientific, Waltham, MA) supplemented with proteinase inhibitor cocktail (Roche catalogue #04693159001; 7x stock solution), according to manufacturer's instructions. 60 µg of protein was loaded onto 10% SDS-PAGE gel and then transferred onto a PVDF membrane (Millipore, Billerica, MA). The membrane was blocked with 5% milk for 0.5 h and then incubated with GR N499 primary antibody (3 µg/µl, generously provided by Dr. Keith Yammamoto, University of California, San Francisco, 1∶2500 concentration in 2.0% milk) overnight at 4°C. Following incubation, the membrane was washed three rimes 10 min in 10 ml TBST (Tris Base Solution containing 0.1% Tween 20), incubated in secondary antibody (HRP conjugated goat anti rabbit IgG, Santa Cruz Biotechnology, 1∶6000 concentration in 2% milk) for 1.5 h, and washed three times 10 min in TBST. The blot was then developed using Super-Signal West Pico Chemiluminescent Substrate (Thermo Scientific, Waltham, MA) and Kodak Biomax Light Film according to manufacturer's directions.

In order to control loading efficiency, blots were stripped, re-blocked with 5% milk and then incubated in primary goat beta actin antibody at 1∶400 dilution in 2% milk for 2.0 h, washed three times 10 min in TBST, incubated in HRP conjugated donkey anti goat IgG (Sabta Cruz Biotechnology, 1∶5000 dilution in 2% milk) for 1.5 h, and washed three times 10 min in TBST. Following antibody applications and washes, the membrane was developed according to the procedure described above.

### Chromatin Immunoprecipitation Assay (ChIP)

PVN-derived IVB cells were incubated at a density of 10^6^ cells/15 cm plate and were treated with vehicle, 100 µM Dexamethosone (DEX, positive control) or 100 mM EtOH for 2 h. ChIP assay was performed using EZ-Magna ChIP G Chromatin Immunoprecipitation kit (Millipore, Billerica, MA) according to manufacturer's instructions. Briefly, following formaldehyde crosslinking cell lysate was sonicated on wet ice 5X/5 sec using Fisher Scientific Sonic Dimembrator Model 100 and then centrifuged to pellet the debris. The supernatant was collected into 50 µl aliquots and stored in −80°C until further processing. Immunoprecipitation of crosslinked GR-DNA complex was performed by overnight incubation with 12 µg of GR N499 antibody (generously provided by Dr. Keith Yamamoto, University of California, San Francisco) and diluted in ChIP Dilution Buffer supplied in the kit. GR/DNA complexes were reverse crosslinked and the DNA was purified using spin columns provided in the kit. To perform standard end point PCR, CRH promoter specific upper and lower primers (0.25 µM final concentration; 5-TTCTCTCTCCCACTCTGCCTCTTT; 5-TTGGTGACGTCAACGAGCCCTAAA) were added to 5 µl DNA. The PCR product was then analyzed using standard agarose gel electrophoresis. Control GAPDH primers (provided in kit) were used to analyze the loading efficiency (input) in the ChIP reaction.

### In Vitro Toxicology Assay (MTT based)

Cell viability was measured using *in vitro*3-(4,5-Dimethylthiazol-2-yl)-2,5-diphenyltetrazolium bromide) MTT based Toxicology assay (Sigma-Aldrich) according to manufacturer's instructions. Briefly, following EtOH treatments cells were washed once with sterile PBS and media was replaced with 250 µl of a 0.25 mg/ml solution of MTT in regular growth media without FBS and phenol red. Cells were incubated in the MTT solutions for 1.5 h at 37°C; then MTT solution was removed, MTT solubilization solution (200 µl of 0.04 M HCl in absolute isopropanol) was added to each sample and transferred to 96-well plate. Absorbance was read at 570 nm on a multimode multiplate reader (Biotech Inc., Wonooski, VT).

### Statistical Analysis

One-way ANOVA was used to test for differences between specific treatment groups followed by Tukey's post-hoc test if one-way significance was indicated. A Student's t-test was used in order to compare differences between vehicle and EtOH treatment within each deletion mutation. All tests were performed using SigmaStat Statistical Analysis Software. A p-value of less than 0.05 was considered to be significant.

## Results

### EtOH treatment altered CRH promoter activity in a time- dependent manner

**Figure 2:**
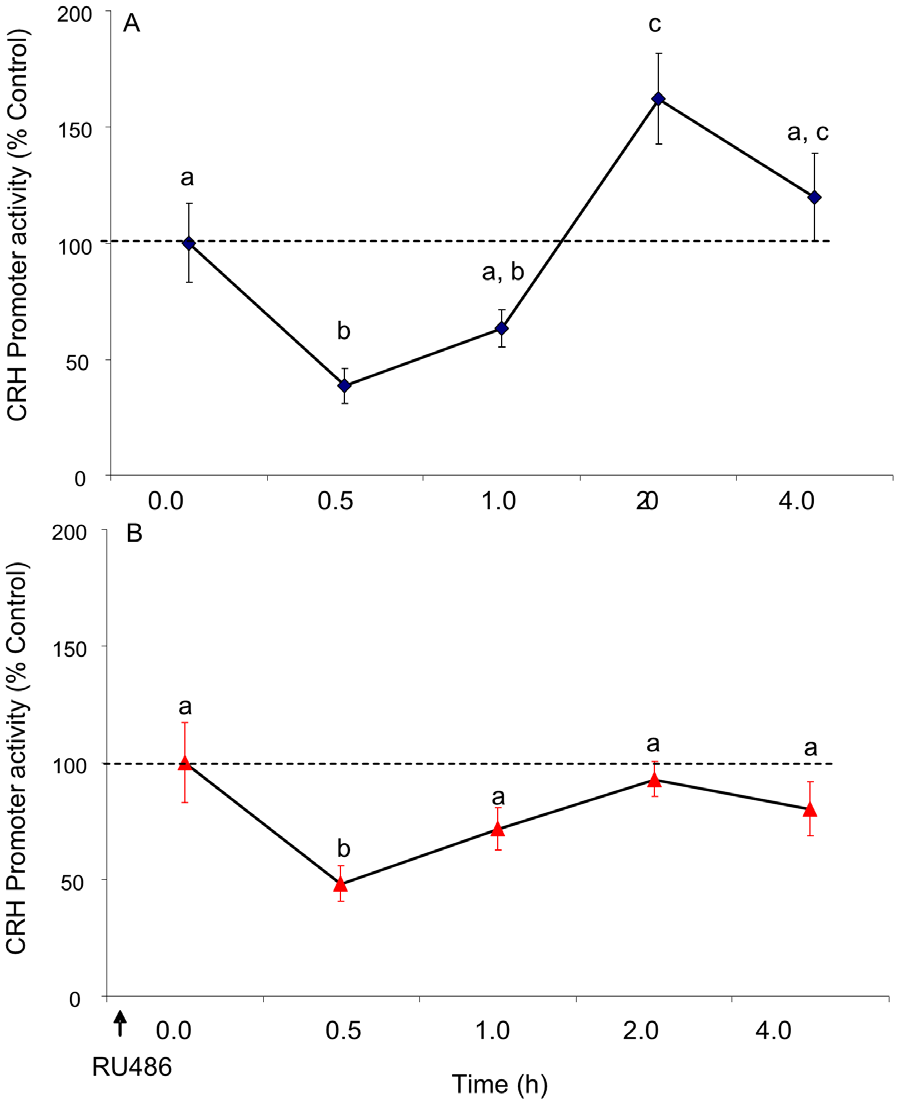
Effects of EtOH treatment (A) and RU486 pre-treatment (B) on CRH promoter activity in a neuronal cell line. CRH-luciferase activity was measured in IVB cell line after treatment with 100 mM EtOH for 0.5, 1.0, 2.0 or 4.0 h or media alone (A) and after 16 h pretreatment with 100 nM RU486 and 100 mM ETOH/100 nM RU486 co-treatment for 0.5, 1.0, 2.0 or 4.0 h (B). Data expressed as % change in luciferase activity of vehicle treated control. Dissimilar letters indicate statistically significant difference (P<0.05).

To determine whether EtOH alters CRH promoter activity, we used an *in vitro* reporter gene assay and added EtOH directly to the cell culture media (rat PVN-derived cell line (IVB). Our results showed that treatment with 100 mM EtOH had a biphasic effect on CRH promoter activity (F(4,28) = 15.331, p<0.001, N = 6, Fig. 2A). Notably, EtOH significantly decreased CRH promoter activity after 0.5 h (p = 0.002), whereas EtOH significantly increased the promoter activity after 2.0 h (p = 0.043). We then treated the cells with EtOH in the presence of a glucocorticoid receptor antagonist (RU486) in order to ascertain whether EtOH interacted with glucocorticoid negative feedback mechanisms at the level of the CRH promoter. Treatment with RU486 completely abolished the EtOH-induced increase in CRH promoter activity observed after 2.0 h of EtOH treatment, suggesting that EtOH might interact with glucocorticoid receptors to alter CRH promoter activity. Interestingly, treatment with RU486 did not affect the EtOH-induced decrease in CRH promoter activity observed after 0.5 h of EtOH treatment (Fig. 2B).

### EtOH increased CRH promoter activity at 2.0 h time point at all doses tested

**Figure 3:**
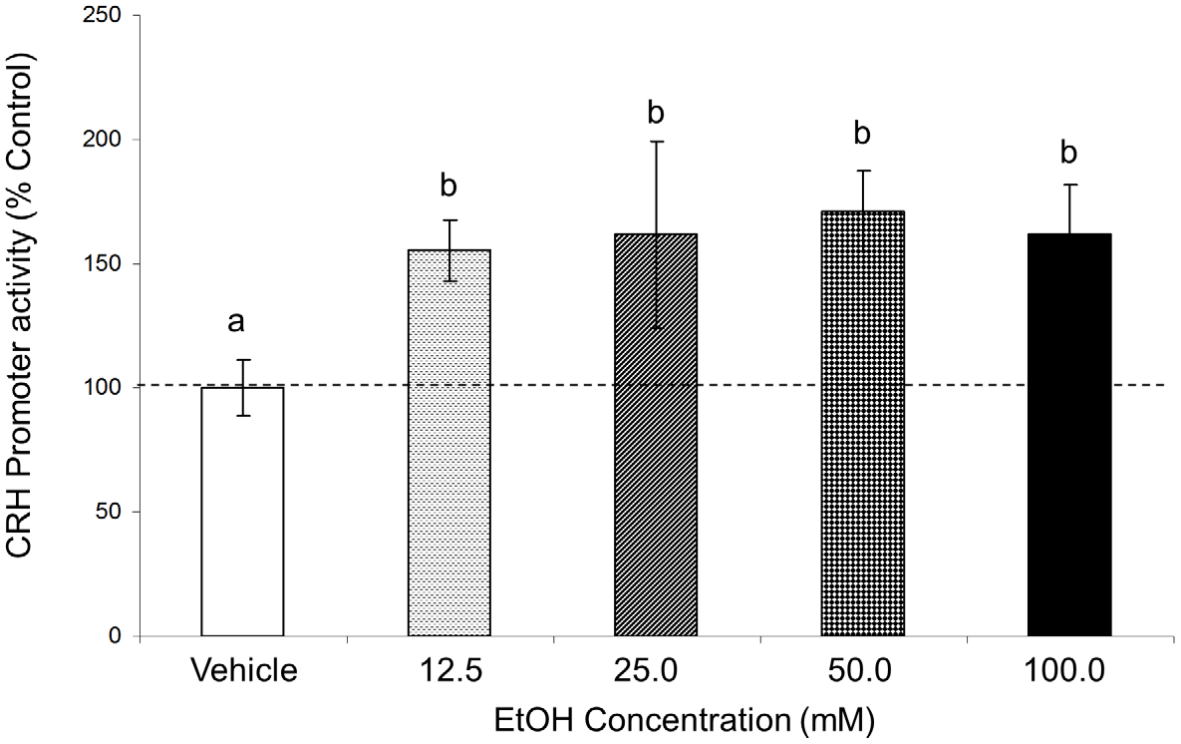
Effects of 2.0 h EtOH treatment with different doses of EtOH on CRH promoter activity. CRH-luciferase activity was measured in IVB cell line after treatment with 12.5, 25.0, 50.0 and 100 mM EtOH for 2.0 h or media alone Data expressed as % change in luciferase activity of vehicle treated control. Dissimilar letters indicate statistically significant difference (P<0.05).

The results obtained from initial experiments in Figure 2 demonstrated that EtOH alters CRH promoter activity. The dose used in those experiments (100 mM) was based on previously published studies that used EtOH treatment in a cell culture model system however, the physiological relevance of this high dose is questionable given that the blood alcohol level corresponding to the legal limit for driving (0.08%) is equivalent to about 25 mM [13], [14], [15], [16], [17], [18]. Therefore, we tested CRH promoter activity in the presence of varying doses of EtOH in order to establish the minimal effective dose. The highest dose (100 mM) increased CRH promoter activity to the same degree following 2.0 h of EtOH treatment as observed in the previous time course experiments (Figs. 2A and 3). Surprisingly, however, EtOH significantly increased CRH promoter activity at all of the lower doses we tested compared to vehicle treated control (F(4,45) = 2.665, p = 0.044, N = 6, Fig. 3). Also, there was no dose response observed, suggesting that lowest 12.5 mM dose is sufficient to elicit a maximal response of CRH promoter activity. Thus, for all subsequent studies the 12.5 mM dose was used in order to be consistent with doses that were more physiologically relevant.

### Deletion of GR binding sites within nGRE abolished the alcohol induced increase in CRH promoter activity at 2.0 h

**Figure 4:**
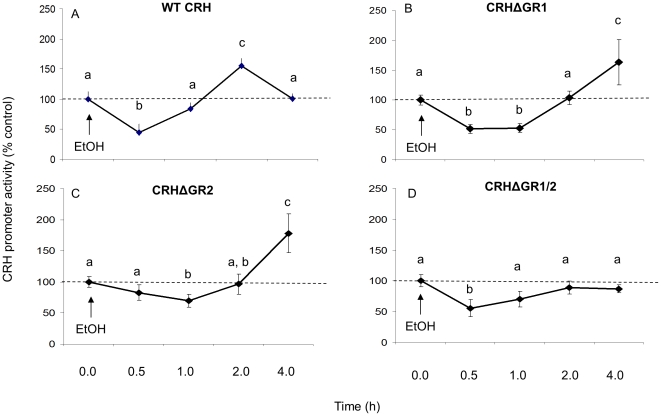
Effects of EtOH treatment on WT and mutated CRH promoter activity. Luciferase activity was measured in IVB cell line after treatment with 12.5 mM EtOH for 0.5, 1.0, 2.0 or 4.0 h or vehicle in A) WT CRH promoter, B) promoter lacking GR binding site 1 (CRHΔGR1), C) GR binding site 2 (CRHΔGR2) or D) GR binding site 1 and 2 (CRHΔGR1/2). Data expressed as % change in luciferase activity from vehicle treated control. Dissimilar letters indicate statistically significant difference (P<0.05).

Our initial observations using the GR antagonist RU486 suggested that EtOH might alter CRH promoter activity by interfering with GR and normal glucocorticoid negative feedback pathways. We screened the promoter sequence and identified two GR binding sites located proximal to the transcription start site. Site-directed mutagenesis was performed to delete each site individually (CRHΔGR1 and CRHΔGR2) or concurrently (CRHΔGR1/2). One-way ANOVA indicated that the GR binding site deletions had a significant effect on basal CRH promoter activity (F(3, 25) = 25.449, p<0.001, N = 6, Fig. 4). Deletion of CRHΔGR1 and CRHΔGR2 significantly increased basal CRH promoter activity (p<0.001), whereas deletion of both GR binding sites (CRHΔGR1/2) did not affect basal CRH promoter activity (p = 0.551). Deletion of GR binding site 1, 2 or both (CRHΔGR1, CRHΔGR2 or CRHΔGR1/2) abolished the previously observed EtOH-induced increase in CRH promoter activity at the 2.0 h time point (p = 0.764. p = 0.836 and p = 0.457, respectively, Fig. 4), yet did not affect the EtOH-induced decrease in CRH promoter activity at seen at 0.5 h (Fig. 4). Interestingly, individual deletion of GR binding sites 1 or 2 did not abolish the EtOH-induced increase in CRH promoter activity rather, the time course was delayed such that the promoter activity increased after 4 hours of EtOH exposure as opposed to the previously observed increase at 2.0 h (Figs. 2, 3 and 4). EtOH did not induce CRH promoter activity in the double deletion mutant (CRHΔGR1/2) even after 8 hours of EtOH exposure (data not shown).

**Figure 5:**
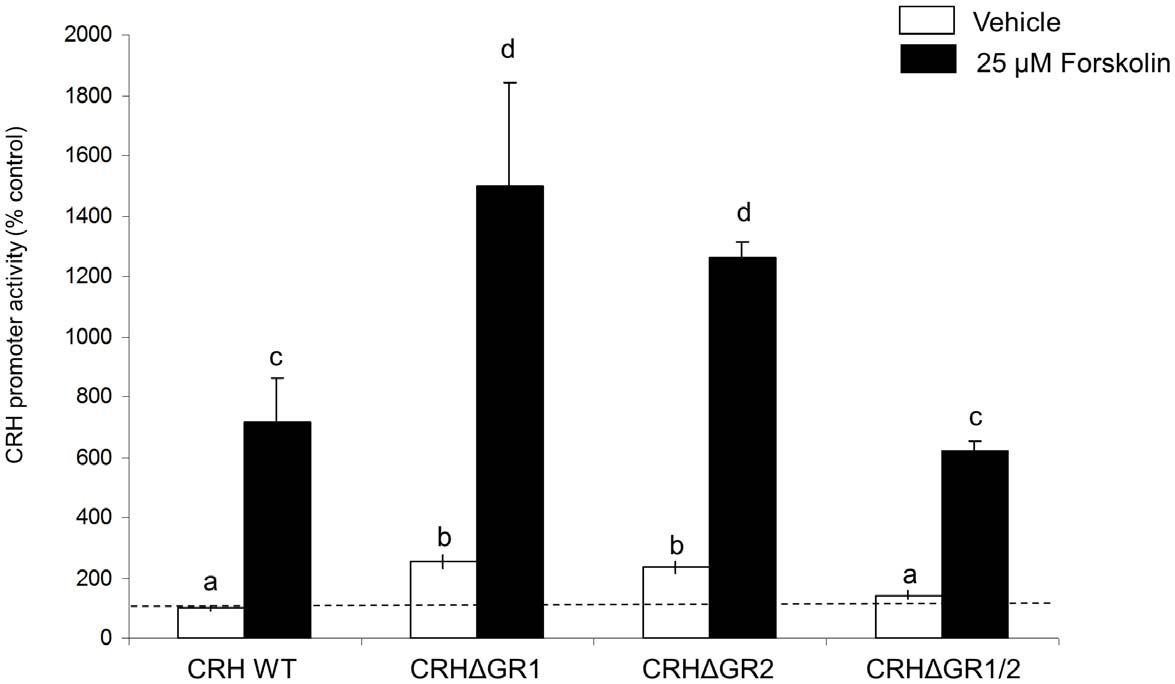
Effects of Forskolin treatment on WT and mutated CRH promoter activity. Luciferase activity was measured in IVB cell line after treatment with 25 µM Forskolin for 6.0 h (black bars) or vehicle (white bars) in WT CRH promoter or mutated promoter lacking GR binding site 1 (CRHΔGR1), GR binding site 2 (CRHΔGR2) or GR binding site 1 and 2 (CRHΔGR1/2). Data expressed as % change in luciferase activity of vehicle treated control. Dissimilar letters indicate statistically significant difference (P<0.05).

Validation of the mutated CRH promoter constructs was confirmed by treatment with 25 µM forskolin, which is a potent activator the CRH promoter. As expected, forskolin treatment significantly increased CRH promoter activity in all CRH mutant promoter constructs (Fig. 5).

### EtOH treatment did not affect GR mRNA or protein expression in IVB cells

**Figure 6:**
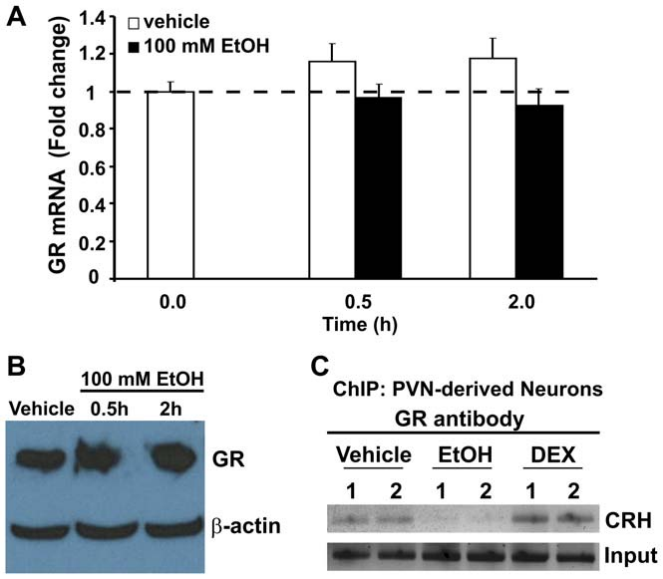
Effects of EtOH treatment on GR mRNA expression, GR protein levels, and GR:nGRE binding. (A) GR mRNA expression in IVB cells were measured after treatment with vehicle (white bars) or 100 mM EtOH for 0.5 or 2.0 h (black bars). Data are expressed as fold change in GR mRNA expression calculated according to the standard ΔΔct method. (B) GR protein was measured in IVB cells after treatment with vehicle (lane 1) or 100 mM EtOH for 0.5 (lane 2) or 2.0 h (lane 3) using Western Blot analysis. Data shown are representative of 3 replicate experiments. (C) IVB cells were treated with vehicle or 100 mM EtOH for 2 h and processed for chromatin immunoprecipation (ChIP) assay using GR antibody and primers flanking the nGRE site on the CRH promoter. PCR products were resolved on a 2% agarose gel. Data shown are representative of 3 replicate experiments.

It is possible that EtOH directly affected GR expression, thereby altering CRH promoter activity. In order to test this we measured GR mRNA and protein expression in IVB cells following treatment with 100 mM EtOH for 0.5 or 2.0 h using qRT-PCR and Western Blot analysis, respectively. Our data revealed that there were no changes in GR mRNA expression in IVB cells relative to baseline (F(4,47) = 0.1.889, p = 0.128) at any time point tested (p = 0.435 and p = 0.082 for 0.5 and 2.0 h, respectively) or protein levels (Fig. 6A, B).

### EtOH treatment precluded GR binding to nGRE on CRH promoter

We next tested whether GR binding to the nGRE on the CRH promoter was hindered in the presence of EtOH using chromatin immunoprecipitation (ChIP) assays. Our results showed a positive association of GR with the nGRE on the CRH promoter in the presence of vehicle (media containing 10% FBS) alone, as expected (Fig. 6C). Treatment with the potent GR agonist, dexamethasone (DEX) appeared to enhance GR association with the chromatin, whereas EtOH exposure completely eliminated GR:chromatin association (Fig. 6C).

### Inhibition of protein kinase A (PKA) blocked EtOH-induced increase in CRH promoter activity

**Figure 7:**
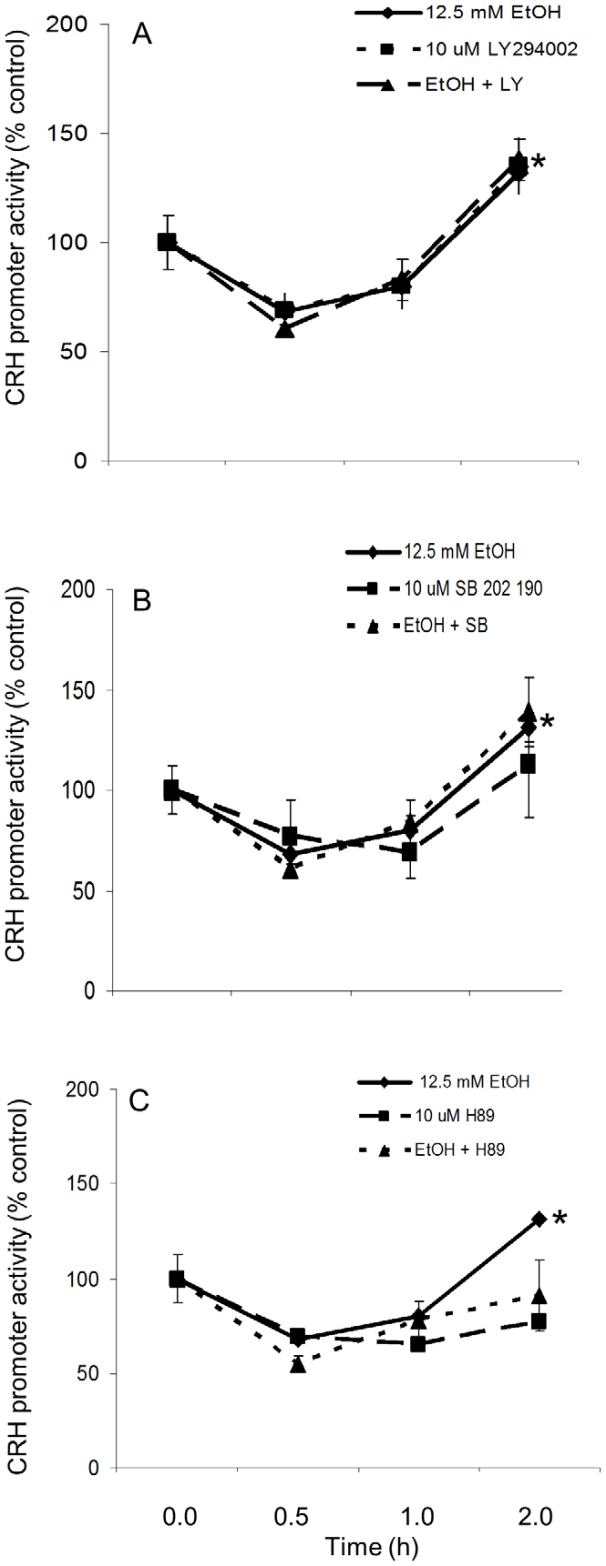
Effects of concomitant EtOH and kinase inhibitor treatments on WT CRH promoter activity. Luciferase activity was measured in IVB cell line after treatment with 12.5 mM EtOH and 10 µM (A) LY294,002 (B) SB 202190 (C) H89 for 0.5, 1.0, 2.0 or 4.0 h. Data expressed as % change in luciferase activity from vehicle treated control. * indicates significant difference compared to control group at 0 h (P<0.05).

Previous studies have demonstrated that inhibition of PKA blocked the EtOH-induced increase in CRH [13]. To verify if PKA is acting similarly in our system, as well as to examine the potential impact of other kinase pathways, we tested the effects of EtOH on CRH promoter activity in the presence of three kinase inhibitors: 1) phosphatidylinositol 3 kinase (PI3K) inhibitor LY 294,190; 2) p38 mitogen activated protein kinase (p38 MAPK) inhibitor SB 202 190; and 3) the PKA inhibitor H89. As shown in Fig. 7, the PKA inhibitor H89, but not LY294,190 and SB 202 190, blocked the EtOH-induced increase in CRH promoter activity at 2.0 h. As expected, EtOH alone significantly increased CRH promoter activity at 2.0 h (p = 0.046). Concomitant treatment with LY 294,002 (p = 0.40 compared to EtOH at 2 h; Fig. 7A) and SB 202 190 (p = 0.42 compared to EtOH at 2 h, Fig. 7B) had no effect on the EtOH-induced increase in CRH promoter activity at 2.0 h, whereas the EtOH effect was blocked by H89 (p = 0.006 compared to EtOH at 2 h, Fig. 7C). Interestingly, inhibition of p38 MAPK with LY 294,002 alone significantly increased CRH promoter activity after 2 h, suggesting that MAPK-mediated phosphorylation events might be important for maintaining baseline levels of CRH promoter activity. Together, these data demonstrate that the PKA pathway is involved in mediating EtOH induced changes in CRH promoter activity.

### 100 mM EtOH treatment did not induce cell death

**Figure 8:**
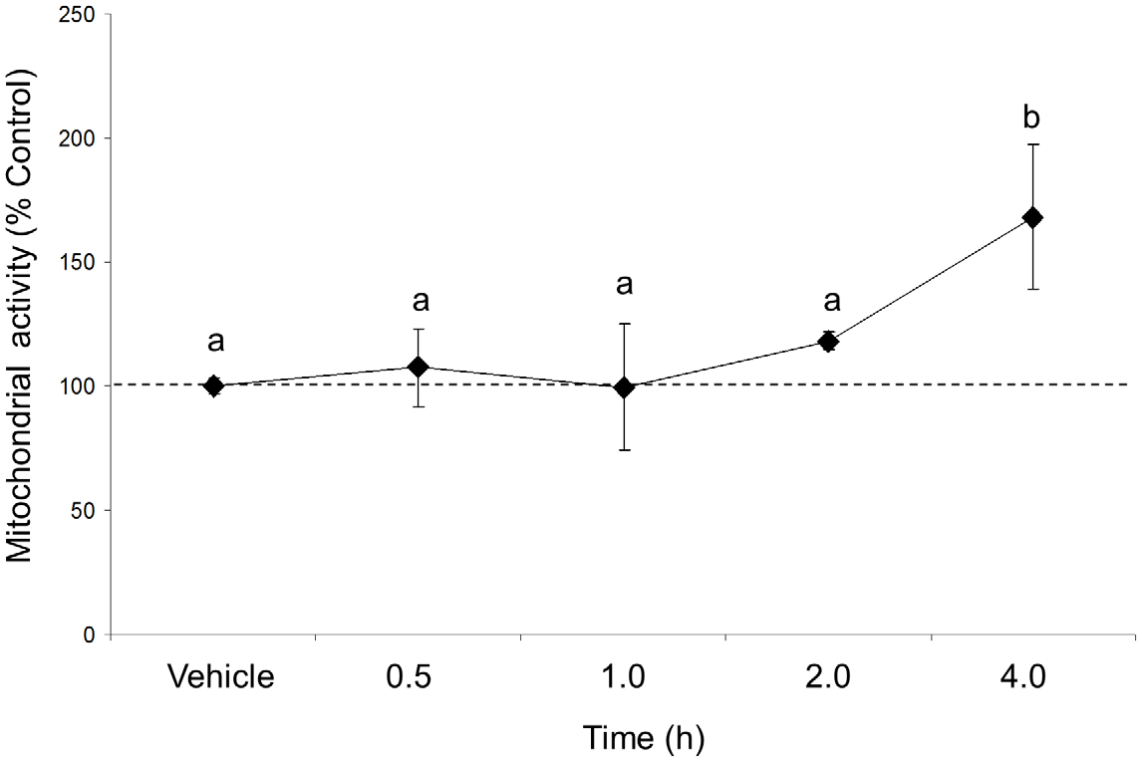
Effects of 100 mM EtOH treatment on cell viability in a neuronal cell line. Mitochondrial activity was measured in IVB cell line after 0.5, 1.0, 2.0 and 4.0 h of 100 mM EtOH treatment. Data expressed as % change of mitochondrial activity relative to vehicle (10% FBS media) treated controls. Dissimilar letters indicate statistically significant difference (P<0.05).

We used a MTT assay to determine whether our highest dose of EtOH treatment (100 mM) induced cell death in our IVB cell line. The results showed that EtOH treatment had a significant effect on cell viability (F(4,10) = 3.495, p = 0.049, N = 3, Fig. 8). Contrary to what would be expected, EtOH did not decrease cell viability at any time point measured, rather, it increased mitochondrial activity at 4.0 h time point.

## Discussion

These studies demonstrate for the first time that very low doses of EtOH (12.5 mM) are sufficient to activate the CRH promoter and, importantly, have identified a specific nucleotide sequence on the CRH promoter that is required for EtOH-mediated activity. Our data showed that EtOH treatment significantly modulates CRH promoter activity in a time-dependent manner, with a significant decrease in promoter activity occurring after 0.5 h of EtOH exposure, followed by a robust (p<0.05) increase after 2 h. Further, the observed increase in promoter activity at 2 h was mediated through an interaction between EtOH and glucocorticoid receptor signaling, as evidenced by a complete abolishment of the EtOH-mediated effect using two methods of GR inhibition: the GR antagonist RU486 and deletion of the GR binding site. Moreover, we showed that GR binding to the nGRE site on the CRH promoter was precluded in the presence of EtOH. Taken together, these data support our hypothesis that alcohol might increase CRH mRNA expression in the PVN *in vivo* by directly interfering with glucocorticoid negative feedback mechanisms exerted at the level of the CRH promoter.

EtOH has previously been shown to increase intracellular levels of the second messenger cAMP [19], [20]. The CRH promoter contains one cAMP-responsive site (CRE) located −224 bp upstream from the transcription start site. Importantly, the CRE site lies in close proximity to the nGRE that we targeted in our studies, and these two sites have been shown to act cooperatively to modulate normal CRH promoter activity [21]. For instance, when ligand-bound GRs bind to the nGRE they prevent signaling of cAMP response element binding protein (CREB) at the CRE site of the CRH promoter thereby, decreasing the activity of the promoter and subsequent transcription of CRH mRNA. Based on our data, we propose that GR-CRH promoter interaction at the nGRE site is blocked in the presence of EtOH allowing for free access of phosphorylated CREB to bind the CRE site and thereby, activate the promoter. Evidence supporting the importance of the CRE binding site was demonstrated by Li et al., (2005) who showed that an EtOH-induced increase in CRH promoter activity was prevented by inhibition of protein kinase A (PKA), a downstream target of the cAMP signaling pathway [13]. We replicated these results and also showed that inhibition of the PI3K and p38 MAPK pathways did not interfere with EtOH's ability to increase CRH promoter activity, suggesting that EtOH effects on CRH are specifically mediated by PKA. Despite differences in cell lines (hybrid rat neuroblastoma/mouse glioma vs. PVN-derived) and CRH promoter length (1500 vs. 2100 bp), our results are consistent with those of Li et al., demonstrating EtOH-induced increases in CRH promoter activity after 2 h of exposure. Taken together, these studies provide strong evidence that the CRE and nGRE sites on the CRH promoter are both important and might act cooperatively to mediate EtOH action.

Chronic EtOH treatment has been shown to alter GRE:DNA binding in rat cortex and hippocampus. Adult rats fed a chronic alcohol diet (Lieber-deCarli, 15 days) had decreased GRE:DNA binding, as measured by gel electromobility shift assays, that was fully restored after 72 hours of EtOH withdrawal [22]. Further, they showed an EtOH-mediated decrease in GR protein levels in the cortex and hippocampus, which was likely associated with the observed overall decrease in GR:DNA binding in those same regions [22]. In our study, we specifically targeted the nGRE located on the CRH promoter as a potential site for EtOH-mediated modulation of CRH promoter activity and showed that GR binding to the nGRE on the CRH promoter was precluded in the presence of EtOH, although it is important to note that our results cannot exclude the possibility that EtOH might have also prevented GR protein:protein interactions with CREB or other proteins in the larger CREB complex, thereby blocking GR occupancy at the CRE site as well. Moreover, deletion of portions of this site on the CRH promoter abolished EtOH-induced changes in the promoter activity. Deletion of individual sites (CRHΔGR1 or CRHΔGR2) abolished the EtOH-induced increase at 2 h however, a significant increase was observed after 4 h suggesting that EtOH impeded, but did not entirely prevent, normal GR binding. By contrast, concurrent deletion of both GR binding sites abolished all EtOH-induced increases in CRH promoter activity, similar to what was observed in the wild type CRH promoter following concomitant EtOH treatment with the GR antagonist, RU486.

Contrary to the results obtained by Roy, et al., we did not observe any EtOH-mediated changes in GR mRNA or protein expression [22]. One possibility is that GR protein levels change with chronic, as opposed to acute, EtOH treatment, although Spencer and McEwan [23] also noted that there were no changes in cytosolic fractions of mineralocorticoid receptor (MR) or GR following chronic EtOH treatment in the brains of adult rats [23]. By contrast, Little at al., (2008) showed that chronic EtOH treatment (28 week, liquid diet) and 2 week withdrawal increased nuclear GR protein but did not change the cytosolic GR protein levels in the prefrontal cortex of male C57BL mice [24]. In our study we measured GR mRNA and GR protein and did not see any changes following 2 h of EtOH exposure in a PVN-derived cell line, suggesting that the discrepancies in the reported literature might be due to differential effects of EtOH on GR expression in different brain regions.

The findings herein have furthered our understanding of the molecular mechanisms of EtOH-induced activation the HPA axis by demonstrating that the nGRE site is a critical component for the observed EtOH-mediated increases in CRH promoter activity. However, we also observed a significant decrease in CRH promoter activity after 0.5 h of EtOH exposure that was not explained by cell death or inhibition of GR. The EtOH-induced decrease in promoter activity following 0.5 h of exposure persisted in the presence of the GR antagonist RU486, and with all mutant GR-binding site deletion constructs, suggesting that very short EtOH exposures do not interfere with normal negative feedback mechanisms. The mechanism for a rapid EtOH-induced decrease in CRH promoter activity is still unknown and is being actively investigated in our ongoing studies.

Overall, results presented in this study show that in a PVN-derived neuronal cell line, alcohol increased CRH promoter activity by interfering with GR signaling at the nGRE site of the promoter, providing a potential molecular mechanism by which EtOH treatment *in vivo* increases CRH mRNA levels in the PVN.
